# Impact of multisession 40Hz tACS on hippocampal perfusion in patients with Alzheimer’s disease

**DOI:** 10.1186/s13195-021-00922-4

**Published:** 2021-12-20

**Authors:** Giulia Sprugnoli, Fanny Munsch, Davide Cappon, Rachel Paciorek, Joanna Macone, Ann Connor, Georges El Fakhri, Ricardo Salvador, Giulio Ruffini, Kevin Donohoe, Mouhsin M. Shafi, Daniel Press, David C. Alsop, Alvaro Pascual Leone, Emiliano Santarnecchi

**Affiliations:** 1 Berenson-Allen Center for Non-Invasive Brain Stimulation, Beth Israel Deaconess Medical Center, Harvard Medical School, Boston, MA USA; 2 Department of Radiology, University Hospital of Parma, Parma, Italy; 3 Department of Radiology, Beth Israel Deaconess Medical Center, Boston, MA USA; 4 Center for Advanced Medical Imaging Sciences, Division of Nuclear Medicine and Molecular Imaging, Department of Radiology, Massachusetts General Hospital, Harvard Medical School, Boston, MA USA; 5 Neuroelectrics, Barcelona, Spain; 6 Hinda and Arthur Marcus Institute for Aging Research and Deanna and Sidney Wolk Center for Memory Health, Hebrew Senior Life, Boston, MA USA; 7 Department of Neurology, Harvard Medical School, Boston, MA USA; 8 Guttmann Brain Health Institute, Barcelona, Spain; 9 Precision Neuroscience & Neuromodulation Program, Gordon Center for Medical Imaging, Massachusetts General Hospital, Harvard Medical School, Boston, MA USA

**Keywords:** Dementia, Cerebral blood flow, Neuromodulation, Neurostimulation, tES, Gamma band, Gamma activity, Hippocampus, EEG, CBF

## Abstract

**Background:**

Alzheimer’s disease (AD) is associated with alterations in cortical perfusion that correlate with cognitive impairment. Recently, neural activity in the gamma band has been identified as a driver of arteriolar vasomotion while, on the other hand, gamma activity induction on preclinical models of AD has been shown to promote protein clearance and cognitive protection.

**Methods:**

In two open-label studies, we assessed the possibility to modulate cerebral perfusion in 15 mild to moderate AD participants via 40Hz (gamma) transcranial alternating current stimulation (tACS) administered 1 h daily for 2 or 4 weeks, primarily targeting the temporal lobe. Perfusion-sensitive MRI scans were acquired at baseline and right after the intervention, along with electrophysiological recording and cognitive assessments.

**Results:**

No serious adverse effects were reported by any of the participants. Arterial spin labeling MRI revealed a significant increase in blood perfusion in the bilateral temporal lobes after the tACS treatment. Moreover, perfusion changes displayed a positive correlation with changes in episodic memory and spectral power changes in the gamma band.

**Conclusions:**

Results suggest 40Hz tACS should be further investigated in larger placebo-controlled trials as a safe, non-invasive countermeasure to increase fast brain oscillatory activity and increase perfusion in critical brain areas in AD patients.

**Trial registration:**

Studies were registered separately on ClinicalTrials.gov (NCT03290326, registered on September 21, 2017; NCT03412604, registered on January 26, 2018).

**Supplementary Information:**

The online version contains supplementary material available at 10.1186/s13195-021-00922-4.

## Background

Alzheimer’s disease (AD) is the most common cause of dementia, and its prevalence continues to increase [1]. Despite this enormous disease burden and intensive scientific research, therapeutic options are limited. While there are pharmacologic interventions that transiently stabilize cognitive function, no disease-modifying therapy is available. Even promising pharmacological interventions (e.g., aducanumab, an anti-amyloid compound) do not appear to stop cognitive decline [2]. In an effort to develop effective treatments, research has then focused on deepening our understanding of the pathophysiology of AD. Positron emission tomography (PET) as well as single-photon emission computed tomography (SPECT) imaging revealed marked hypometabolism and perfusion deficits in AD patients with respect to healthy controls [3]. More recently, a perfusion-sensitive MRI imaging sequence, arterial spin labeling (ASL), has been developed to study brain perfusion without the need for contrasting agents [4]. ASL has helped reveal a significant reduction of brain perfusion (cerebral blood flow - CBF) in the temporal, parietal, and posterior cingulate cortices in AD patients with respect to healthy subjects [3, 5], even though a pathological blood flow increase in preclinical stages of the disease has also been described [6, 7]. Moreover, CBF reduction has been correlated with language impairment in AD patients [8], and its reduction is paralleled by disease progression starting from the prodromal stage [9]. Also, hypoperfusion may predict conversion to AD in mild cognitive impairment (MCI) patients [10], making the quest for approaches to modulate (i.e., increase) perfusion a priority.

A recent preclinical animal study has shown that gamma (γ) band oscillatory brain activity is directly responsible for arteriolar vasodilatation and the consequent increase in blood oxygenation [11]. Gamma activity usually refers to cortical oscillations in the 30–80Hz frequency band, primarily generated by the interaction between inhibitory interneurons such as parvalbumin (PV)+ interneurons, and pyramidal cells [12]. The investigators found that optogenetic manipulations of γ-band electrical power entrain the vasomotor oscillations of corresponding cortical and penetrating arterioles in a unidirectional way, i.e., independently from baseline CBF [11]. In turn, fluctuations in arteriolar diameter coherently drive fluctuations in blood oxygenation [11]. Electrical activity drives the arteriolar vasomotion with a lag of 2 s, which in turn leads to changes in functional MRI signal (detecting increases of oxygenation in the venular component; BOLD signal) one second later [11]. The significance of this electro-arteriolar coupling has been related to the role of vasomotion in the removal of waste and toxic proteins via the paravascular space, i.e., the so-called glymphatic system, that seems to be impaired in AD mouse models and AD patients [13, 14].

Pivotal preclinical studies have also shown that exogenously-induced increase of gamma oscillations (specifically at 40Hz) promotes microglial activation and cause subsequent reduction of Aβ and p-tau depositions in a mouse model of AD [15]. Decreased γ activity in AD mouse models is linked to PV+ inhibitory interneuron pathology that interferes with fast inhibitory loops in cortical circuits and is associated with a hyperactivation of pyramidal cells leading to global network dysfunction [16]. Remarkably, induction of gamma activity in presymptomatic AD mice—and thus a restoration of the physiological activity of PV interneurons—prevents subsequent neurodegeneration and behavioral deficits [17]. As seen in preclinical models [18], a consistent finding in patients with AD is a relative attenuation and dysregulation of gamma activity [19], therefore gamma induction may represent a novel and powerful therapeutic approach [17].

Recently, a neuromodulation technique that delivers alternating current stimulation—transcranial alternating current stimulation (tACS)—has received attention for the possibility of translating the aforementioned animal evidence to humans via noninvasive induction of gamma activity [20]. tACS applies low-amplitude alternating (sinusoidal) current to enhance specific oscillations by entraining neurons under specific cortical rhythms, depending on the applied stimulation frequency (e.g., 40Hz) [21]. Non-human animal work has demonstrated that tACS entrains neurons in widespread cortical areas [22], with recent non-human primates experiments revealing dose-dependent neural entrainment and increased burstiness as the fundamental response to tACS [23, 24]. Simulations, supported by empirical evidence using electroencephalography (EEG), demonstrated that tACS modulates brain oscillatory activity via network resonance, suggesting that a weak stimulation at a resonant frequency could cause large-scale modulation of network activity [25], and amplify endogenous network oscillations in a frequency-specific manner [18]. Consequently, tACS has been found able to modulate brain oscillation and related behavior in healthy subjects and patients [26–28], with an enhancement of gamma oscillations via tACS leading to transient improvement in motor, working memory, and abstract reasoning tasks on healthy controls [29–31], and effects often lasting beyond the tACS application period [30, 32, 33].

Given the evidence of impaired gamma activity in AD patients, and the potential of restoring brain perfusion via gamma entrainment, in the present pilot studies, we aimed to translate to humans the aforementioned preclinical findings on 40Hz gamma stimulation in animal models of AD by means of tACS applied to a sample of 15 mild to moderate AD patients. We hypothesize that a multiday course of tACS would lead to an increase in CBF in regions targeted by tACS, with a stronger effect for participants receiving longer tACS treatments. Additionally, we hypothesized that CBF changes would show some spatial specificity in relation to the different tACS electrode montages used in the studies, and potentially covariation with changes in the spectral power of gamma as measured via EEG as well as episodic memory scores indexing temporal lobe/hippocampal function.

## Methods

### Participants and experimental design

Fifteen participants with mild to moderate dementia due to AD were enrolled in total (mean age 72 years, male = 9; Mini-Mental State Examination - MMSE = 23.53, SD = 3.35). Participants were enrolled in two separate open-label clinical trials exploring the impact of different tACS doses (i.e., number of stimulation sessions) and targeting approaches (i.e., positioning of tACS electrodes on the scalp and resulting induced electrical field in the brain). Participants received 1 h of daily tACS for 2 or 4 weeks in hospital settings (Monday to Friday), with baseline (pre-tACS) and follow-up (post-tACS) assessments composed of cognitive and memory testing, EEG, and perfusion MRI (ASL) data. Participants underwent additional assessments pre/post tACS not reported in the present manuscript and beyond the scope of the present study, e.g., PET imaging for Aβ and p-tau, transcranial magnetic stimulation (TMS) measures, combined TMS-EEG recording, voice biomarkers recording, blood biomarkers.

**Fig. 1:**
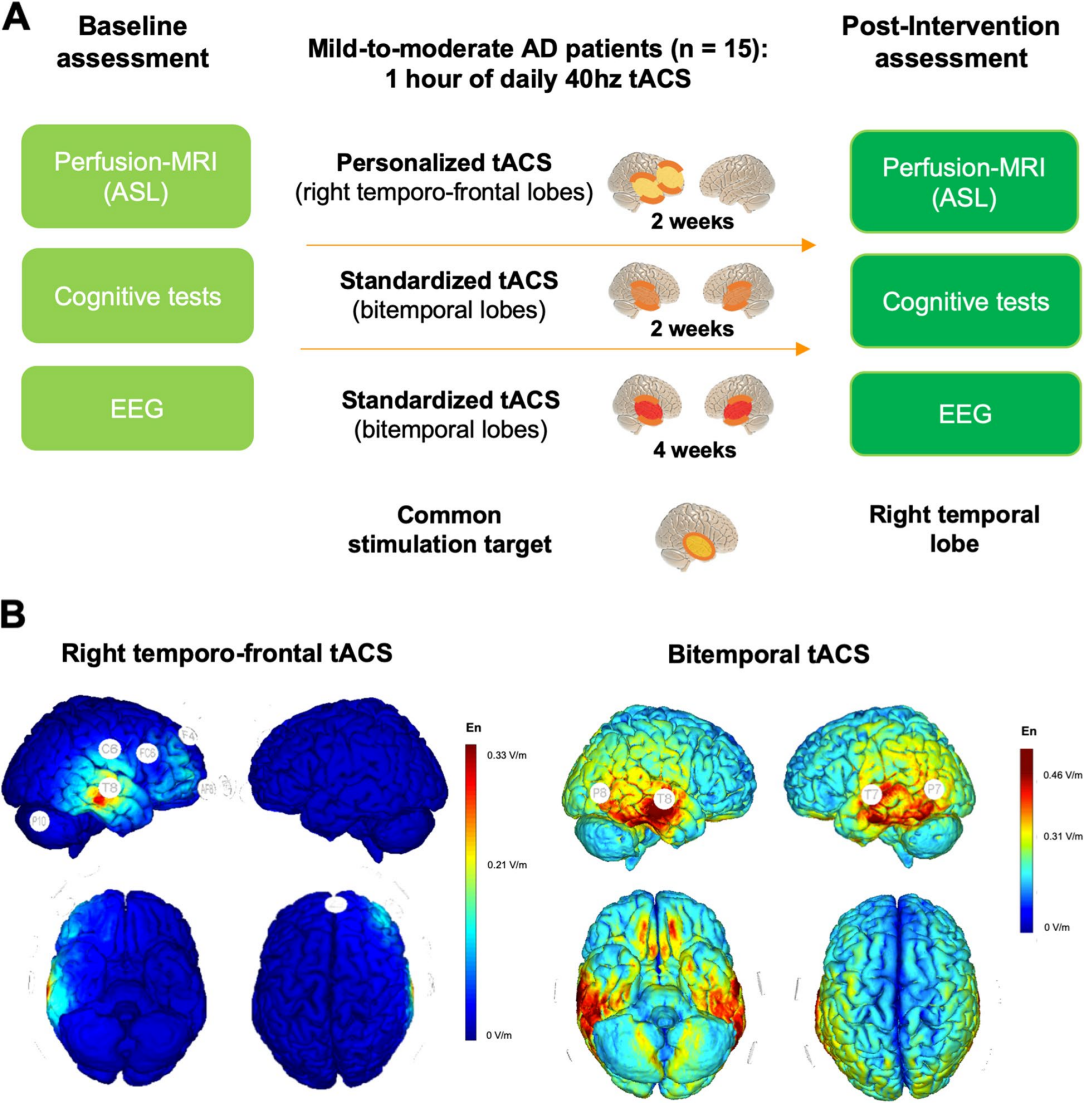
Experimental protocol. **A** Study design and relevant pre-post tACS measures. Fifteen participants with mild to moderate dementia due to AD were enrolled in total (mean age 72 years, male = 9; MMSE = 23.53, SD = 3.35). Participants received 1 h of daily tACS for 2 or 4 weeks in hospital settings (Monday to Friday), with baseline (pre-tACS) and follow-up (post-tACS) assessments composed of cognitive and memory testing, EEG, and perfusion MRI (ASL) data. Participants underwent additional assessments pre/post tACS not reported in the present manuscript and beyond the scope of the present study, e.g. PET imaging for Aβ and p-tau, transcranial magnetic stimulation (TMS) measures, combined TMS-EEG recording, voice biomarkers recording, blood biomarkers. tACS was conducted targeting the normal component of the electric field either to the bilateral temporal lobes (bitemporal tACS hereafter) or unilateral (right) temporal and frontal lobes (temporo-frontal tACS hereafter), thus always impacting the right temporal lobe across all participants (corresponding to T8 on the 10/20 EEG system). Therefore, participants can be subdivided into three subgroups: (i) subjects receiving 2 weeks (10 sessions = 10h) of unilateral temporo-frontal tACS (Group 1; *n*=5); (ii) subjects receiving 2 weeks (10 sessions = 10h) of bitemporal tACS (Group 2; *n*=5); (iii) subjects receiving 4 weeks (20 sessions = 20h) of bitemporal tACS (Group 3; *n*=5). Common site of stimulation across montages was represented by the right temporal lobe. **B** On the left, normal electrical field (En-field) for representative subject receiving unilateral temporo-frontal tACS (Group 1), on the right En-field for participants receiving bilateral temporal lobe stimulation (Groups 2 and 3)

Depending on the tACS paradigms, participants can be subdivided into three subgroups: (i) subjects receiving 2 weeks (10 sessions = 10h) of unilateral temporo-frontal tACS (Group 1; *n*=5); (ii) subjects receiving 2 weeks (10 sessions = 10h) of bitemporal tACS (Group 2; *n*=5); (iii) subjects receiving 4 weeks (20 sessions = 20h) of bitemporal tACS (Group 3; *n*=5) (Fig. 1). Common site of stimulation across montages was represented by the right temporal lobe (Fig. 1). Within a 1-week period before and after the tACS intervention, participants underwent a cognitive assessment battery, 64 channels scalp EEG, and MRI assessments. All participants gave written informed consent prior to participating in the studies, registered separately on ClinicalTrials.gov (NCT03412604, NCT03290326; PI Santarnecchi).

The first pilot trial NCT03290326 was designed to assess safety and feasibility of performing a two-weeks stimulation tACS treatment in patients with AD. Among the first 10 patients enrolled (Groups 1 and 2 in the manuscript), Group 1 underwent a personalized right-sided stimulation centered on the temporo-frontal lobes. The rationale was to personalize stimulation on the basis of individual Aβ accumulation maps based on Florbetapir PET imaging. In Group 1 participants, regions with higher Aβ load on the right temporal and frontal lobes were targeted, with stronger tACS intensity over the temporal lobe given the higher load of Aβ compared to the frontal lobe (Fig. 1). This approach led to Group 1 subjects receiving stimulation in the right temporo-frontal lobes; however, the electrodes of maximal injected current were slightly different across participant on the basis of the distribution of their amyloid load (i.e., maximal current density on EEG electrode positions F4 and T8 in patient X, F2 and T8 in patient Y). The unilateral personalized stimulation was conceived as a method to (i) test the spatial specificity of tACS stimulation on the individual target regions in the AD brain, (ii) improve the localization and/or recognition of ictal/EEG changes during and/or after the treatment (i.e., more probable in the stimulated right hemisphere). Given that in the first 5 patients (Group 1) a very good spatial localization was achieved and no epileptiform alterations were detected at the electrophysiological as well as clinical assessments, the investigators decided to stimulate Group 2 bilaterally, specifically over the temporal lobes.

Bitemporal tACS was conceived as a way to induce more focal stimulation over the temporal lobes, given the typical Aβ and tau protein distribution in the AD brain [34]. Additionally, tau protein, particularly expressed in the temporal lobe, is also significantly correlated with cognitive decline in AD patients, compared to a weak to null association for Aβ. Therefore, more emphasis on the temporal lobe would guarantee a higher chance of potentially modulating tau accumulation in the future, while also still targeting hypoperfusion present in the same area in AD patients. Finally, in the NCT03412604 trial corresponding to Group 3, bitemporal stimulation was conducted for 4 weeks to enhance the probability of inducing perfusion and protein changes, while at the same time testing the safety and feasibility of a 4-weeks stimulation protocol. The research proposal and associated methodologies were approved by the local ethics committee (Beth Israel Deaconess Medical Center IRB) in accordance with the principles of the Declaration of Helsinki.

### Transcranial alternating stimulation (tACS)

tACS was delivered via a battery-driven current stimulator (Starstim SS32, Neuroelectrics, Barcelona, Cambridge) through surface circular Ø 20mm PISTIM electrodes (Neuroelectrics, Barcelona, Spain) with an Ag/AgCl core and a gel/skin contact area of 3.14 cm^2^. The electrodes were placed into holes of a neoprene cap corresponding to the international 10/20 EEG system. Gel (Signa, PARKER LABORATORIES, INC.) was applied to optimize signal conductivity and lower impedance. Electrode impedance was checked before starting each tACS session to assure safety and maximal efficacy of stimulation as well as to ensure familiarization of participants with the tACS-induced scalp sensations (e.g., tingling). For all sessions, 32 electrodes were placed on the scalp to record EEG before and after each tACS session, although only a subset of the electrodes was used to deliver tACS [35]. tACS at a stimulation frequency of 40Hz was applied for 1 h with a maximum intensity of 2mA on each electrode and 4mA total across all electrodes, preceded by a 30-s ramp up period and followed by a 30-s ramp down period, while research and clinical personnel carefully monitored for side effects for the entire duration of each session. Common site of stimulation across all patients and montages was represented by the right temporal lobe targeted via T8 (10/20 EEG system). Given the long stimulation sessions and the specific patient population, during tACS participants were instructed to watch a series of pre-selected videoclips from a list of selected documentaries freely available on YouTube, with the aim of maintaining a constant brain state while reducing distraction and avoiding constant interaction with operators in the room. The research team selected videos based on their length (i.e., to be approximately 1 h long), thematic subject (i.e., excluding documentaries related to war or other conflictual subjects that could cause excessive arousal and activation in the participants), and language (i.e., excluding those with extremely technical/specific terms). The themes were counterbalanced across genres to provide a nice selection of videos that would engage participants and focused their attention (primary goal of the videoclips) and be palatable for patients with diverse preferences (e.g., documentaries on animals, nature, history, technology, as well as on filmmaking and music). Each day patients were offered to choose from the list, or resume the video presented during the previous tACS session.

### Biophysical modeling

Given the expected variability in cortical atrophy among participants, we did not use a fixed montage (electrode positions and currents) across participants of Group 1, but instead, we defined a cortical target designed to keep normal electrical field (En-field) amplitude fixed on the highest Aβ deposition areas, seeking to ensure that participants received a similar electric field dose. Some variability was still observed due to the constraints on the currents that the stimulator can output and the different sizes of the targets. The resulting montage included 8 stimulation electrodes, delivering tACS at 40Hz with a maximum intensity of 2mA on each electrode according to current tACS safety guidelines [36], and with a resulting higher induced field on the temporal lobe due to Aβ distribution. In Group 1, tACS was not in-phase for all electrodes since obtaining in-phase only stimulation would not be possible (current conservation). The target phase for each area was optimized so that the induced field would be maximal on the PET-defined targets, leading to an inter-region 180 phase choice. The approach for identifying optimal stimulation targets by fusing PET and MRI data for each patient was developed by the PI of the studies (ES); personalized montages were computed by the PI in collaboration with the Neuroelectrics team using the Stimviewer algorithm and the methods described in [37, 38], adapted to the case of tACS.

The same safety guidelines were followed for the stimulation templates used for Group 2 and Group 3, but stimulation was targeted over the bilateral temporal lobes via 4 fixed stimulating electrodes (P8, T8, P7, and T7, right electrodes delivering current with a 180° phase degree respect to the left ones), given the usual pattern of deposition of Aβ and tau protein commonly involving the bilateral temporal lobes when patients are symptomatic [34] (Fig. 1B). Electrode locations and stimulation intensity for bitemporal tACS used in Groups 2 and 3 were defined by the PI of the studies (ES). Stimulation intensity was titrated for each patient, given the typical discomfort reported for transcranial electrical stimulation (tES) delivered over the temporal regions. Whole-scalp 64-channel resting-state EEG was collected in the week before and during the week after the tACS treatment course, along with a comprehensive neurocognitive assessment.

### MRI scan

Neuroimaging acquisition was performed on a GE 3 Tesla MR750 scanner using a 32-channel head array coil from Nova Medical. Participants underwent high resolution T1-weighted structural scan (3D T1-w BRAVO), two runs of resting state functional connectivity, resting perfusion MRI with ASL, diffusion tensor imaging, T2* GRE, and FLAIR sequences (total scan timing 60 min). ASL studies were performed with 3D pseudo-continuous labeling (1.45s labeling, 2.025s post-labeling delay), background suppression, and 32 centric ordered 4mm thick slices.

### ASL preprocessing and analysis

ASL data preprocessing was performed via an ad-hoc pipeline implemented in MATLAB (MATLAB 2016b, MathWorks) and SPM12 (https://www.fil.ion.ucl.ac.uk/spm/) developed by the laboratory of one of the co-authors at BIDMC (DCA, inventor of the pseudo-continuous ASL technique). Segmentation into three classes (grey matter, white matter, and CSF) and normalization of 3D T1w BRAVO images were obtained via DARTEL [39]. ASL subtraction images were co-registered to the grey matter map and normalized to MNI152 space. Normalized CBF maps were masked with Intracranial Volume (ICV) mask from SPM12 and globally normalized for the CBF values. Manual masking of every CBF map with the corresponding normalized grey matter mask obtained during the segmentation process was performed for each patient for the two timepoints (pre-stimulation and post-stimulation). Grey matter CBF maps were smoothed with a full width at half maximum (FWHM) of 6 mm.

In order to check for perfusion MRI data quality and check that that participants aligned the expected values of CBF in the temporal lobes (usually around 30 mL/min/100g for grey matter in AD patients [3, 5]), individual CBF values from the temporal lobes were extracted using the REX toolbox (https://www.nitrc.org/projects/rex/) embedded in SPM12. Longitudinal statistical analyses assessing the impact of tACS were conducted in SPM12 on normalized grey matter CBF maps via a paired *t* test on all participants (*n* = 15; single voxel level *p*<0.001, cluster-level *p*<0.05, FDR corrected) as well as on each group of participants separately. Analyses were carried out at whole-brain level, to ensure observed changes in CBF were not amplified by the selection of a specific region-of-interest. In the case a significant change in perfusion was identified, CBF changes within significant clusters were correlated with changes (Δ = post minus pre) at episodic memory and language tests, as well as changes in gamma spectral power after tACS measured via EEG. Finally, the SPM Anatomy toolbox was used to label significant clusters extracted via SPM via a probabilistic atlas (see Table 1).

**Table 1:** Significant clusters of changes in perfusion across all subjects. Probability anatomical mapping and cluster coordinates for significant CBF changes detected when analyzing whole-brain cortical CBF changes (post>pre) across all subjects (upper panel), and for Group 3 participants who received the 4 weeks tACS intervention

*All subjects*			Cluster #1		Anatomical probability mapping
Local Maxima Subcluster	*t* value		MNI		
		*x*	*y*	*z*	
#1	t = 4.64	32	18	-40	R Medial Temporal Pole
#2	t = 3.87	28	2	-44	R Fusiform Gyrus
#3	t = 3.18	26	0	-40	R Entorhinal Cortex
#4	t = 2.96	26	8	-36	R Entorhinal Cortex
#5	t = 2.82	26	16	-40	R Medial Temporal Pole
#6	t = 2.69	32	12	-42	R Medial Temporal Pole
#7	t = 2.66	28	10	-42	R Medial Temporal Pole
Group 3			Cluster #1		
#1	t = 14.66	24	6	-40	R Entorhinal Cortex
#2	t = 12.84	28	8	-42	R Medial Temporal Pole
#3	t = 9.36	26	12	-44	R Fusiform Gyrus
#4	t = 8.54	30	10	-46	R Fusiform Gyrus
#5	t = 8.38	20	8	-40	R Medial Temporal Pole
#6	t = 7.67	28	18	-40	R Medial Temporal Pole
Group 3			Cluster #2		
#1	t = 4.83	-30	-2	-34	L Fusiform Gyrus
#2	t = 4.18	-26	-6	-30	L ParaHippocampal Gyrus
#3	t = 3.77	-28	-14	-26	L Subiculum
#4	t = 3.66	-32	-14	-26	L CA1 (Hippocampus)
#5	t = 3.39	-30	-10	-26	L CA1 (Hippocampus)

### EEG recording and analysis

Whole-scalp 64-channel resting-state EEG was collected in the week before and in the week after the tACS treatment course via an actiCHamp EEG amplifier system (Brain Products GmbH). EEG recording was obtained while subjects sat in a semi-reclined armchair. During recordings, participants were instructed to remain quiet with their face muscles relaxed. Given the specific study population, particular care was put into ensuring participants understood the importance of staying still and quiet during recording. Both participant and EEG were monitored for signs of drowsiness, at which point the participant was asked to blink their eyes a few times and reminded to stay awake. Recording was done at a sampling rate of 1Khz and impedances were maintained below 5 kΩ during recording.

Data were preprocessed using EEGLAB 2020 [40], Fieldtrip toolbox for EEG/MEG-analysis (Donders Institute for Brain, Cognition and Behaviour, Radboud University, the Netherlands, see http://fieldtriptoolbox.org), the Brainstorm suite [41], and in-house scripts in Matlab R2017b (MathWorks Inc.). Data were initially reduced into 60 dimensions by using principal component analyses (PCA) to minimize overfitting and noise components. Band pass filter was performed using a forward-backward 4th order Butterwoth filter from 1 to 100Hz, a notch filter between 58 and 62Hz was applied, and the data were subsequently referenced to a global average. Subsequently, independent component analysis (ICA) was run to manually remove all remaining artifact components including eye movement/blink, muscle noise (EMG), single electrode noise, cardiac beats (EKG), as well as auditory evoked potentials. Finally, the data were interpolated for missing/removed channels using a 32 spherical interpolation.

Given the longitudinal CBF changes involving primarily the bilateral temporal lobes and the right anterior temporal lobe in particular, changes in gamma spectral power were focused on an array of tACS electrodes indexing the bilateral temporal lobes (i.e., T8, P8, P7, T7), as well as on electrode T8 as a proxy to the right anterior temporal lobe and the common stimulation electrode across tACS montages. Moreover, considering the documented slowing of EEG activity in AD patients [16, 42], with increasing spectral power for activity in the theta and delta band associated with a decrease of fast oscillations such as beta and gamma, statistical analysis was centered on detecting potential changes in gamma spectral power as well as signs of a change in spectral frequency distribution (e.g., restoration of gamma and/or decrease of slower oscillatory activity). Also, considering the limited sample size and exploratory nature of the study, we opted for a simpler statistical framework rather than a full-blown repeated measured ANOVA. Specifically, longitudinal changes in each frequency band were quantified by subtracting baseline absolute spectral power values from post-tACS ones (e.g., baseline theta minus post-tACS theta). A one-way ANOVA with a single factor “Frequency” was computed comparing the pre-posts differences in each frequency band (alpha level = 0.05). Once a main effect was found, post hoc comparisons between pairs of frequency bands were computed as well. EEG bands were defined as follows: delta (1–4 Hz), theta (4–8 Hz), alpha (9–13 Hz), beta (14–30 Hz), low gamma (35–45 Hz), narrow gamma (38–42Hz; centered around the stimulation frequency of 40Hz), mid gamma (45–60 Hz), and high gamma (60–90 Hz). One participant of Group 2 did not complete the post-tACS EEG assessment; analyses were conducted on 14 participants.

### Cognitive assessment

Participants underwent specific tests evaluating global cognition (Alzheimer’s Disease Assessment Scale-Cognitive Subscale (ADAS-cog) [43]; MMSE [44], Montreal Cognitive Assessment (MoCA) [45], activities of daily living (ADL) [46]) to assess any potential change in overall cognitive functioning after tACS. Additionally, tasks assessing cognitive functions relevant for the brain regions stimulated by tACS were also used, using the National Alzheimer’s Coordinating Center Uniform Data Set (NACC UDS) Neuropsychological Battery: the Craft Story 21 Recall Immediate and Delayed addressing episodic memory [47], and the Category Fluency task (animals), a widely used measure of verbal fluency and language [48].

## Results

All 15 participants completed the study and tolerated the intervention with only minor side effects commonly reported in the tACS literature: tingling (10/15) rated as mild; scalp irritation (7/15) rated as mild-moderate; visual changes (8/15) rated as mild-moderate, and headache (5/15, rated as mild-moderate) induced by mechanical pressure from the stimulation cap. Participants attended 95% of the study visits (190/200 daily tACS visits, 10 sessions in total missed distributed across 7 patients), showing excellent treatment compliance. No epileptiform alterations were detected at the electrophysiological as well as clinical assessments.

### Perfusion changes

Mean CBF values in the right temporal lobe calculated at baseline (pre tACS intervention) across all the participants were 32.3 mL/min/100g (SD = 6.8), consistent with literature on hypoperfusion in AD patients [3, 5] and validating image acquisition and CBF extraction procedures. Post intervention CBF values in the right temporal lobe increased significantly from 32.3 to 34 mL/min/100g (SD = 8.5) (*t* = 2.01, *p* < 0.05; Cohen’s *d* = 0.22). CBF values of the left temporal lobe were extracted for patients of Group 2 and 3 (*n*=10) who received bitemporal tACS, showing a mean value of 33 mL/min/100g (SD = 6.5) at baseline and 34 mL/min/100g (SD = 6.4) after the intervention (*t* = 1.24, *p* = 0.11). CBF values of the right frontal lobe were extracted for Group 1 that received personalized right temporo-frontal stimulation, revealing a baseline CBF of 35.5 mL/min/100g (SD = 9.7) and 39 mL/min/100g (SD = 14) after the intervention (*t* =2.36, *p* = 0.35; Cohen’s *d* = 0.29).

**Fig. 2:**
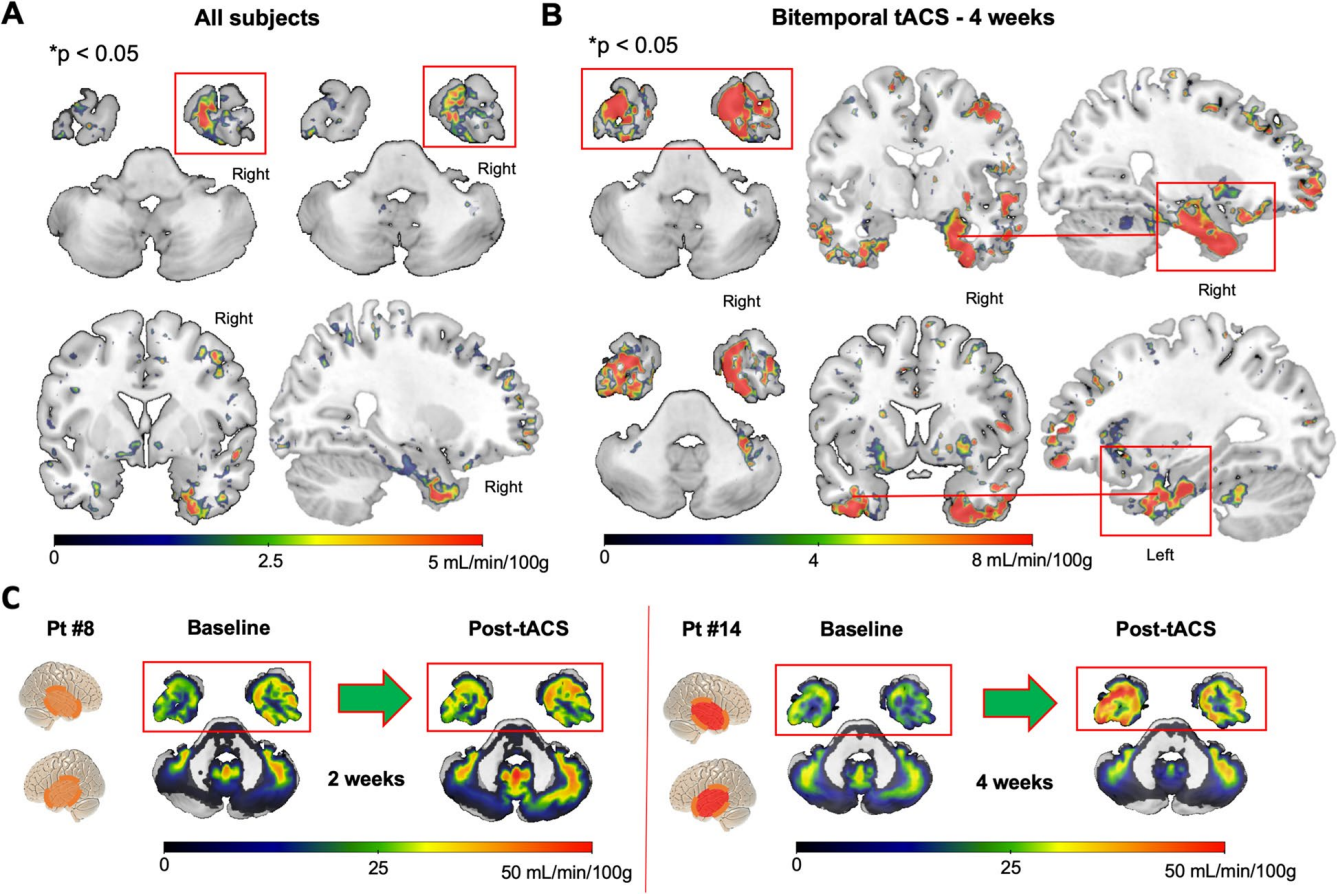
Perfusion results. **A** CBF increase after tACS. Paired t test (post>pre, *p*<0.05, FDR-corrected) revealed an increase of CBF selectively involving the right temporal lobe, representing the common site of stimulation across participants (*n* = 15). **B** Whole cortical brain CBF analyses of participants who received the highest dose of tACS (20h of bilateral temporal lobe stimulation over 4 weeks, *n* = 5, Group 3) revealed a selective increase in CBF in the bilateral temporal lobes, accordingly to the stimulation template (*p*<0.05, FDR-corrected). **C** Examples of CBF variations in two representative participants belonging to Group 2 (pt #8) and 3 (pt #14)

Apart from standard regional CBF assessment, voxel-wise whole brain analyses with no prespecified masks were performed to guarantee a more unbiased result. When comparing post-tACS CBF maps with pre-tACS CBF maps across the entire brain in all subjects, a significant CBF increase was detected in multiple anatomical clusters primarily located in the right temporal lobe (*n*=15, Fig. 2A) (*p*<0.05, FDR-corrected), specifically involving the right medial temporal pole, fusiform gyrus, and entorhinal cortex (see Table 1 for probability anatomical mapping and clusters’ coordinate). Of note, results are consistent with the right temporal lobe being the only region consistently stimulated across all 15 participants.

**Fig. 3:**
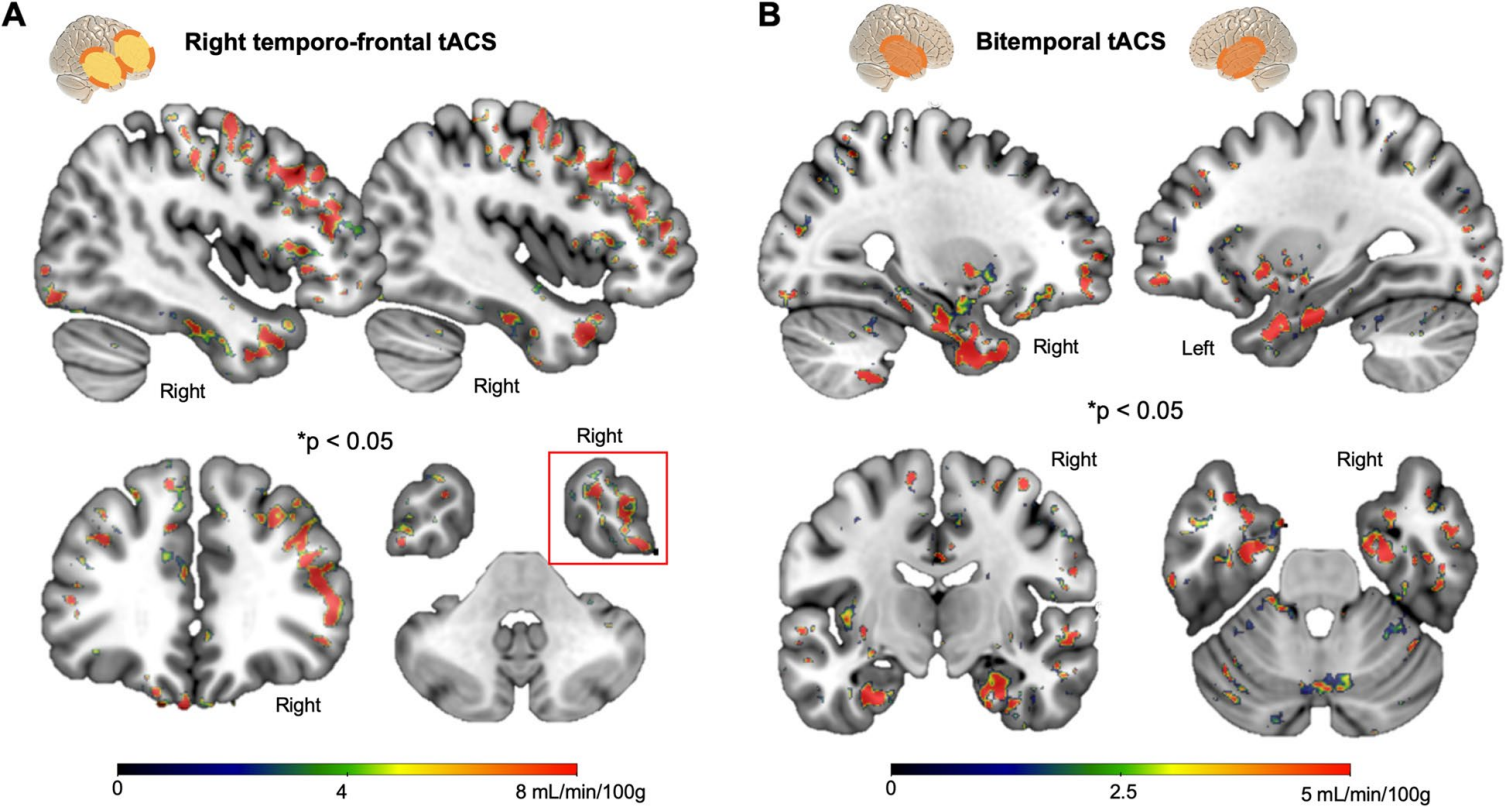
Comparison of CBF increases between tACS montages. **A** When looking at significant CBF increase after tACS in Group #1 of participants who received right temporo-frontal stimulation, a pattern of predominant right temporo-frontal CBF increases was found, matching with tACS targeting. **B** CBF increase in participants from Group #2 + #3 (*n* = 10) who received bilateral temporal lobe stimulation showed a significant increase of CBF predominantly localized in bilateral temporal regions

Dividing participants on the basis of their respective tACS montage and looking at pre-post tACS CBF changes, both temporo-frontal (Group 1) and bitemporal (Groups 2 and 3) tACS montages resulted in significant CBF changes, however displaying different topographies of local CBF increase reflecting the two montages: Group 1 (right temporo-frontal tACS; Fig. 3A); Group 2 − 3 (bitemporal tACS; Fig. 3B).

Additionally, when looking specifically at Group 3 who received the longest intervention targeting the bilateral temporal lobes (i.e., 20 h of tACS over 4 weeks compared to 10h in Group 1 and 2), a significant pattern of CBF increase mainly involving the bilateral temporal lobes was found, including medial temporal poles, fusiform gyri, bilateral entorhinal cortices and hippocampi (see Table 1 for probability anatomical mapping and clusters’ coordinate), matching the bitemporal tACS montage (*t* = 2.13, *p*<0.05, FDR-corrected; Fig. 2B). For more detailed results on CBF changes in the left and right temporal lobe please see Supplementary Materials.

### Post-tACS EEG changes

**Fig. 4:**
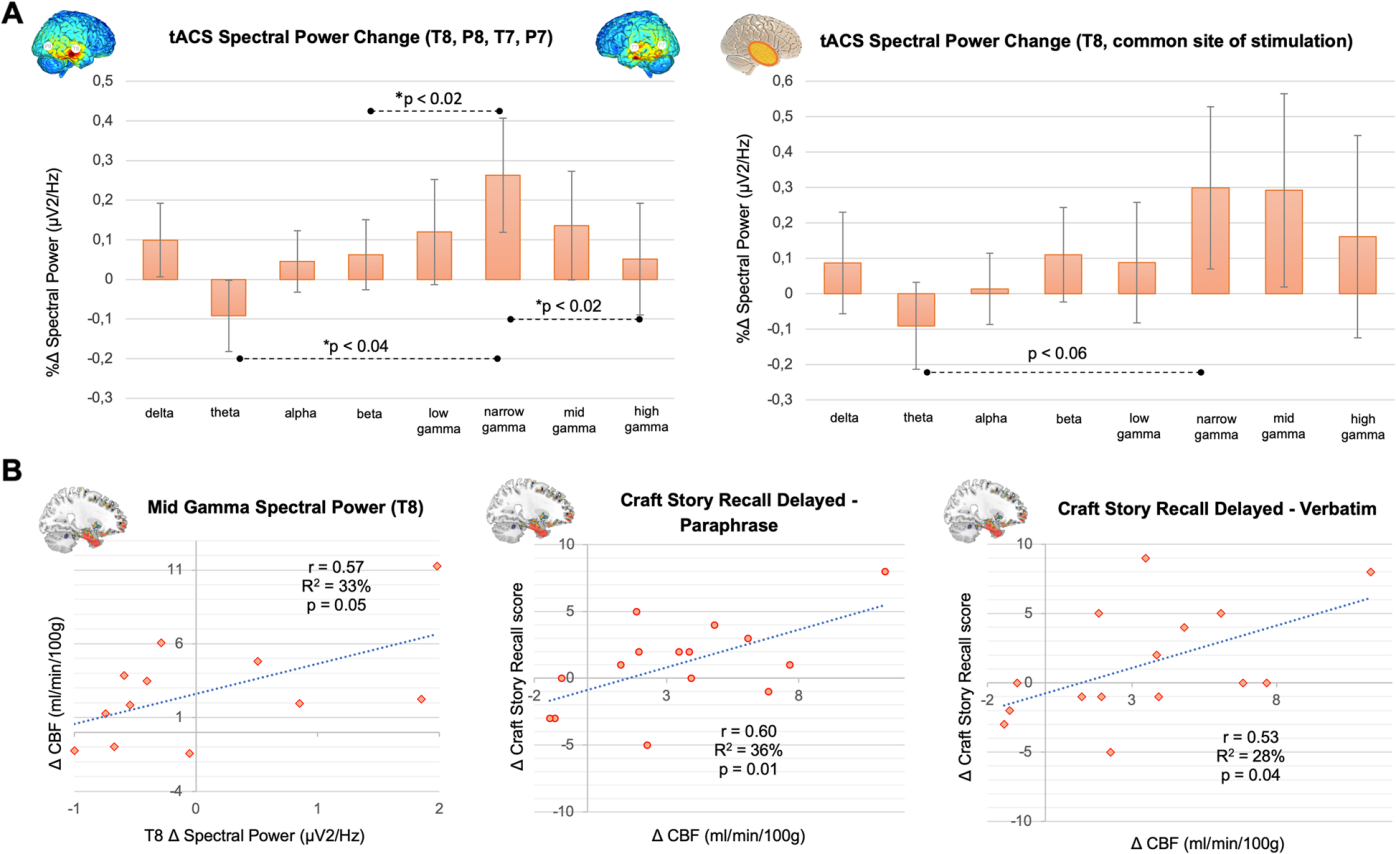
CBF changes, EEG results and correlations with cognition. **A** Changes in spectral power in the gamma band for a cluster of EEG electrodes indexing regions displaying post-tACS increase in perfusion (T8, P8, P7, T7; left panel) are reported, as well as for the electrode T8 (right panel) representing the common tACS electrode across all participants and the scalp electrode more proximal to the right anterior temporal lobe displaying the highest change in CBF post-tACS. **B** Spectral power changes in the narrow gamma band (38-42 Hz) detected on T8 significantly correlate with CBF changes in the right temporal lobe (left panel). Significant CBF variations also showed a significant correlation with variations in memory performance scores pre-post tACS. Specifically, CBF variation in the right temporal regions across all participants (*n*=15) positively correlated with performance changes at both paraphrase (mid panel) and verbatim (right panel) recollection components of an episodic memory task

The One-way ANOVA on T7/8-P7/8 cluster, corresponding to the bitemporal stimulation, revealed an effect of tACS on spectral power around the stimulation frequency (i.e., narrow gamma, 38–42Hz), compared to the rest of the spectrum (*F*_(7,78)_ = 4.12, *p* < 0.05; *η*2 = 0.03) (Fig. 4). Post hoc analysis showed that narrow gamma spectral power displays a post tACS increase higher than activity in the theta band (*t* = 3.43, *p* < 0.01 Cohen’s *d* = 0.031), beta band (*t* = 2.37, *p* < 0.05; Cohen’s *d* = 0.026) and high gamma band (*t* = 1.84, *p* < 0.05 Cohen’s *d* = 0.024) (Fig. 4A). The same analysis performed on electrode T8, the common site of stimulation across groups, produced a similar distribution of pre-post tACS changes across frequencies, with a significant difference between post tACS changes in the narrow gamma and theta bands (*t* = 2.06, *p* < 0.05; Cohen’s d 0.025). Even though other gamma frequency sub-bands displayed a similar trend of narrow gamma, no other comparisons reached significance.

Narrow gamma spectral power changes observed on T8 were found to significantly correlate with increase in CBF in the right anterior temporal lobe. In details, narrow gamma spectral power changes on T8 significantly correlated with the cluster of significant CBF increase extracted among all available participants (total = 12; one participant did not complete the post tACS EEG assessment, 2 outliers were removed) (*r* = 0.57; *p* = 0.05; *R*^2^= 33% Fig. 4B).

### Cognition and perfusion longitudinal correlation

No significant changes (*p* > 0.05) in overall cognition were found after tACS (ADAS-Cog baseline mean = 18.27, SD = 7.68, post = 18.11, SD = 7.69, Cohen’s *d* = 0.02; ADL baseline mean = 68.5, SD = 4.68, post = 68.3, SD = 6.23, Cohen’s *d* = 0.03; MMSE baseline mean = 23.53, SD = 3.35, post = 22.77, SD: 3.68, Cohen’s *d* = 0.21; MoCA: baseline mean = 15.73, SD = 4.23, post = 17.53, SD: 4.5, Cohen’s *d* = 0.41). Changes at memory and language tests did not reach significance (*p* > 0.05): Craft Story Recall - Immediate (mean pre-tACS *Verbatim* = 8.13, SD = 3.71; mean post-tACS = 7.93, SD = 5.83, Cohen’s *d* = 0.04), and *Paraphrase* (mean pre-tACS Paraphrase = 6.93, SD = 3.77; mean post-tACS = 6.80, SD = 4.1, Cohen’s *d* = 0.03); Craft Story Recall - Delayed (mean pre-tACS *Verbatim* = 3.87, SD = 3.64, mean post-tACS = 5.20, SD = 5.43, Cohen’s *d* = 0.28; mean pre-tACS *Paraphrase* = 3.87, SD = 3.35, mean post-tACS = 4.93, SD = 4.3, Cohen’s d = 0.27); Category Fluency (animals, total correct, mean pre-tACS = 11.47, SD = 5.43; mean post-tACS = 11.33, SD = 4.6, Cohen’s *d* = 0.02).

Significant CBF changes positively correlated with changes at Craft Story Recall - Delayed (Fig. 4C). In detail, ΔCBF value (post tACS CBF minus pre tACS CBF) extracted from the regions displaying a significant longitudinal CBF change after tACS showed a positive correlation with changes (post minus pre) at Craft Story Recall - Delayed Verbatim (*r* = 0.53, *p* = 0.04, *R*^2^ = 0.29) and Paraphrase (*r* = 0.60, *p* = 0.01, *R*^2^ = 0.36) (Fig. 4C). Additional correlations with memory/fluency scores using CBF values extracted separately from the left and right temporal lobes are reported in the Supplementary Materials.

## Discussion

Our data suggest that repetitive sessions of gamma tACS lead to a significant increase in CBF in the temporal lobes, without adverse effects. Specifically, when analyzing whole-brain cortical CBF across all participants, a significant increase was revealed in the right temporal lobe, the region consistently stimulated across all participants, also including the entorhinal cortex (Fig. 2). Moreover, when restricting the analysis to participants receiving bilateral temporal lobe stimulation for 4 weeks (the highest tACS dose), a significant increase in CBF was observed in both left and right temporal lobes, including their mesial parts as well as the hippocampi (Fig. 2). Preliminary evidence of tACS target engagement specificity was observed when comparing perfusion changes obtained with two different stimulation templates (Fig. 3). Finally, gamma spectral power changes were found to be correlated to CBF increase (Fig. 4), as well as moderately correlated with changes in cognitive performance related to episodic memory and fluency (Fig. 4 and Figure S1), two domains loading on the temporal lobe and commonly impaired in AD patients. Findings open up potentially interesting avenues for AD patients and other conditions characterized by hypoperfusion—even though the causal role of hypometabolisms/perfusion in the AD pathophysiological cascade is still unclear—as well as for potential effects related to recent preclinical evidence of gamma-mediated amyloid and tau clearance.

To the best of our knowledge, only two studies have reported an increase in regional CBF in AD patients receiving a drug treatment (e.g., donepezil) [49, 50]. Present preliminary results support the recent growing relevance of gamma activity in AD pathophysiology, while at the same time offering a potential evidence of the recently documented causal role of gamma band activity on vessel diameter variation in the human brain [11]. While gamma activity has been discovered to be a driver of arteriolar vasomotion in animals, the molecular/biological mechanisms translating neuronal spiking into arteriolar diameter variations have not been univocally clarified yet [11]. The most recent observations coming from studies investigating tES in preclinical models suggest the possibility to induce (i) an immediate, primary vasodilatory response via perivascular neurons-mediated and endothelial-mediated pathways (e.g., acting on dural/pial arteries and penetrating arterioles targeted by current flowing in perivascular parasympathetic nerves that mainly cause vasodilatation), and (ii) an indirect, secondary vascular effect via neurovascular coupling, with the involvement of astrocytes and neurons [51–53]. Of note, tES could cause both primary and secondary response by acting on the same target. For instance, pericytes—the cells wrapped around the endothelial wall of capillaries contributing to form the neurovascular unit—regulate the arteriolar and capillary diameter vessels in response to regional neuronal activity [54]. It could be possible that tACS also leads to an indirect modulation of pericytes, as well as astrocytes, as a consequence of direct neuronal modulation, apart from a modulation of peptides released by the stimulated cells themselves [51, 55].

At the pathophysiological level, CBF variations are a consequence of changes in brain glucose metabolism, and a decrease in CBF is thought to reflect synaptic failure [56–58]. Indeed, loss of synapses is considered the most important and direct phenomenon underpinning cognitive decline, ultimately responsible for network disruption [16, 59]. Within this framework, 40Hz tACS could be tackling the AD pathophysiological cascade by modulating interneuron activity contributing to global network dysfunction and by activating microglia waste removal [16], and/or by restoring perfusion in impaired cortical areas to guarantee an adequate amount of nutrients and clearance of toxic products, also given the arteriolar contribution to the glymphatic system pathway [14]. Interestingly, tau pathology has been recently associated with hypoperfusion in the entorhinal cortex, even if the exact underlying pathophysiological mechanism remains to be clarified [60]. Finally, other neuropsychiatric diseases share the cellular substrates of impaired metabolism and reduced interneuron activity observed in AD, in particular frontotemporal dementia (FTD) [16], schizophrenia [61], and autism spectrum disorder [62], suggesting tACS could also benefit these patient populations (e.g., see NCT04425148, 40Hz tACS in FTD).

As for the location of CBF changes and dose-response effects of tACS, good spatial specificity was observed in our data with a primary involvement of the temporal lobes. Previous studies have revealed that alterations in gamma activity over the entorhinal-hippocampal circuit in AD mouse models cause memory impairments [63], and there is ample evidence that gamma and theta oscillations, as well as their phase reciprocal relationship, are crucial for memory processes in general [64]. In particular, gamma oscillations are prominently and physiologically expressed by the entorhinal-hippocampal circuit, potentially making the probability of inducing gamma entrainment via tACS more plausible in these regions, even in the presence of an underlying pathologically desynchronized gamma activity [63]. Further studies with larger samples of participants are needed to identify the most optimal treatment protocols in terms of dose-response effects (i.e., 1 week of daily stimulation followed by rest, 4 weeks of continuative treatment). It must be noticed that high resolution 64-channels EEG recording was performed as part of the baseline and follow-up assessments taking place before and after the entire treatment course. Specifically, the two study visits were prioritized so that they would happen right before (i.e., on a Friday before starting the tACS treatment on the subsequent Monday) and right after the treatment (i.e., on a Monday after the last week of treatment). However, logistical issues related to scheduling of the remaining study visits (e.g., MRI, PET), as well as patients’ availability, sometimes interfered with the originally planned schedule. Therefore, a delay assessment of gamma oscillatory activity post-treatment was present, making the observed changes in gamma spectral power more likely to represent a hint to long-lasting tACS after effects rather than acute changes in brain oscillatory activity. Future studies should include a longitudinal EEG assessment covering multiple time points starting from the end of the last tACS sessions in order to properly characterize individual trajectory of tACS effects.

Even though short as compared to drug trials, the tACS treatment was longer than any publicly available protocol in AD patients or healthy controls—with a maximum of 20h of stimulation over 4 weeks, thus corroborating the safety profile of tACS as well as its feasibility in patients with AD, with no adverse events and strong adherence to the treatment regimen. At the same time, the relatively short duration of the intervention with respect to pharmacological trials (e.g., 6–12 months [65]) could be responsible for the lack of significant changes on global scales of cognition after the intervention, along with the limited sample size. Indeed, a trend for improvement at the MoCA test, known to be able to detect subtle cognitive changes especially in the mild dementia phase, was found (baseline mean = 15.73, SD = 4.23, post = 17.53, SD: 4.5), with a moderate effect size (Cohen’s d = 0.41). This result, along with the observed correlations between post-tACS perfusion and episodic memory changes, support the need for longer trials with a bigger sample size to properly evaluate the potential therapeutical effects of tACS and disentangle the relationship between changes in gamma activity, brain perfusion, and cognitive performance. Home-based tACS delivery should also be considered [66], promoting accessibility to patients and lower burden for caregivers.

Finally, in the present trials, we focused on patients with mild to moderate dementia due to AD given their documented profile of gamma alterations, hypoperfusion, and proteinopathy, allowing to observe potential effects of tACS. However, recent evidence shows how approximately 15–20 years before the onset of cognitive deficits, amyloid starts accumulating, followed by microgliosis and neurofibrillary tangle tau pathology [67], making mild to moderate dementia a relatively advanced stage of AD where significant irreversible neuronal and synaptic loss has occurred and therapeutic countermeasures are likely to be less effective [67]. However, if proven effective, tACS could play a role in earlier stages of the disease, as well as in prodromal AD (e.g., patients with autosomal dominant mutations in the precursor of Aβ or Presenilin), and MCI patients. Given its safety profile and portability, the potential application of tACS as a preclinical preventative intervention aimed at the delay the onset of cognitive manifestations and/or slowing down the course of the disease should be explored.

### Limitations

The trials were intended as pilots and, given the limited sample of participants along with the multiple targeting approaches, did not aim to offer definitive answers on any aspect of the study. A larger sample is needed to confirm the observed increase in CBF as well as changes in gamma spectral power, possibly by reducing the number of daily visits performed at the hospital (e.g., using home-based tACS; present pilots consisted on a total of 200 daily tACS sessions and approximately 240 baseline/follow-ups study visits) and by simplifying the study design in general. A control tACS condition, including sham (placebo) stimulation and potentially a control stimulation frequency, should be included as well (NCT03880240) to ensure that the observed CBF increase is related to the tACS intervention rather than to an unspecific effect of exposure to the study (e.g., daily interaction with health care providers), even if unlikely in the light of the spatial specificity of the effect and the unlikeliness of a spontaneous focal perfusion increase in mild to moderate AD patients.

## Conclusions

Present findings promote a framework for the investigation of tACS-based interventions to increase brain perfusion in AD patients, showing preliminary evidence of the impact of 40Hz tACS on local CBF in the temporal lobe, entorhinal cortex, and hippocampi**.**

## Supplementary Information

**Additional file 1: Supplementary Results**. **Figure S1**. Covariation between changes in temporal CBF and memory and language tasks.

## Data Availability

All data needed to evaluate the conclusions in the paper are present in the paper and/or the Supplementary Materials. Additional data related to this paper may be requested from the authors.

## Abbreviations

AD: Alzheimer’s disease
ADAS-cog: Alzheimer’s Disease Assessment Scale-Cognitive Subscale
ADL: Activities of daily living
ASL: Arterial spin labeling
CBF: Cerebral blood flow
EEG: Electroencephalography
FTD: Frontotemporal dementia
MCI: Mild cognitive impairment
MMSE: Mini-Mental State Examination
MoCA: Montreal Cognitive Assessment
MRI: Magnetic resonance imaging
NACC UDS: National Alzheimer’s Coordinating Center Uniform Data Set Neuropsychological Battery
PET: Positron emission tomography
PV+ interneurons: Parvalbumin positive interneurons
SPECT: Single-photon emission computed tomography
tACS: ranscranial alternating current stimulation (tACS)
