# Supplementary material for: Impact of multisession 40Hz tACS on hippocampal perfusion in patients with Alzheimer’s disease
Source: Alzheimers Res Ther. 2021 Dec 20;13:203. doi: 10.1186/s13195-021-00922-4 (PMC8690894; doi:10.1186/s13195-021-00922-4)
Supplement: Supplementary file 1 — Additional file 1: Supplementary Results. Figure S1. Covariation between changes in temporal CBF and memory and language tasks. [file 13195_2021_922_MOESM1_ESM.docx]

**Impact of multisession 40Hz tACS on hippocampal perfusion in patients with Alzheimer’s Disease**

Giulia Sprugnoli,^1,2^ Fanny Munsch,^3^ Davide Cappon,^1^ Rachel Paciorek,^1^ Joanna Macone,^1^ Ann Connor,^1^ Georges El-Fakhri,^4^ Ricardo Salvador,^5^ Giulio Ruffini,^5^ Kevin Donohoe,^4^ Mouhsin M. Shafi,^1^ Daniel Press,^1^ David C. Alsop,^3^ Alvaro Pascual Leone,^6,7,8^ Emiliano Santarnecchi,^1*^

*Corresponding author.

Email: esantarn@bidmc.harvard.edu

**Supplementary Materials:**

- Supplementary Results
- Fig. S1

Supplementary Results

**Temporal ROIs perfusion correlation with memory and language test scores**

CBF increase in the left temporal lobe was moderately correlated with changes at Category Fluency (r = 0.53, R^2^ = 0.28, p = 0.1) and Craft Story Recall Immediate Paraphrase (r = 0.56, R^2^ = 0.32, p = 0.08) (**Figure S1**, left panel), while right temporal lobe CBF increase showed positive correlations with Craft Story Recall [Delayed Verbatim] (r = 0.53, R^2^ = 0.2, p = 0.04) and [Delayed Paraphrase] (r = 0.58, R^2^ = 0.34, p = 0.02; **Figure S1**, right panel), resembling the correlation observed at whole cortical brain without right temporal lobe masking (**Figure 2D**).


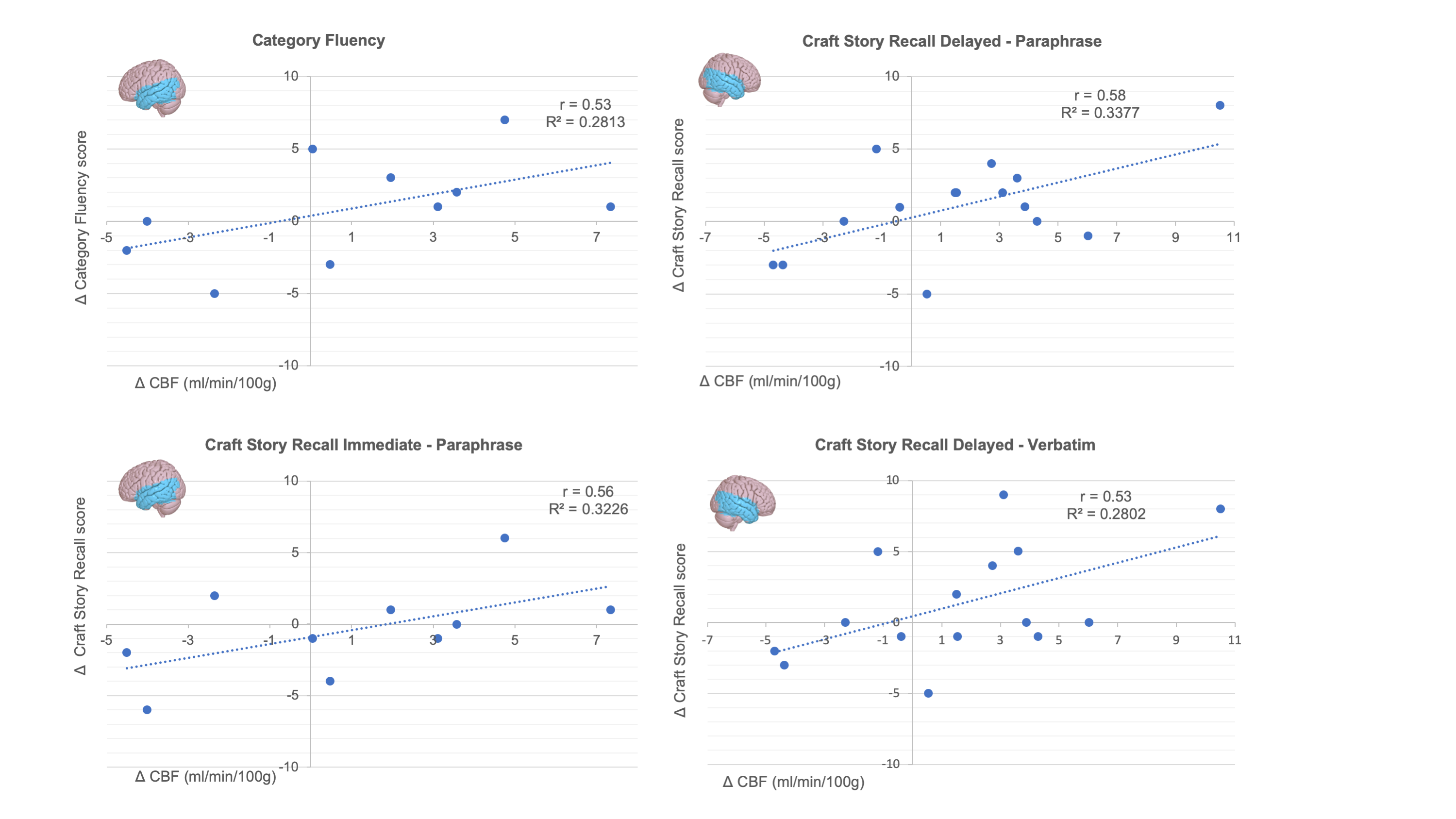


**Fig S1. Covariation between changes in temporal CBF and memory and language tasks.** Longitudinal difference (post minus pre) in CBF values extracted from the left temporal lobe (left panel, for patients receiving tACS over left temporal lobe, n = 10) positively correlate with changes in verbal fluency (i.e., Category Fluency test) and episodic memory (Craft Story Recall test, Immediate – Paraphrase). CBF changes in the right temporal lobe (right panel, for patients receiving tACS over the right left temporal lobe, n = 15) positively correlate with changes in episodic memory performance (Craft Story Recall test, delayed Verbatim and Paraphrase).
